# Lessons from Large-Scale Tolerability and Acceptability Studies of Triple Drug Mass Drug Administration Performed to Support Policy Change and Accelerate Elimination of Lymphatic Filariasis

**DOI:** 10.4269/ajtmh.21-0840

**Published:** 2022-03-15

**Authors:** Gary J. Weil, Peter U. Fischer, Alison Krentel

**Affiliations:** ^1^ Washington University in St. Louis, St. Louis, Missouri;; ^2^ School of Epidemiology and Public Health, University of Ottawa, Ottawa, Canada;; ^3^ Bruyére Research Institute, Ottawa, Canada

## Abstract

Early clinical trials clearly demonstrated the superior efficacy of triple drug therapy with *ivermectin* plus *DEC* and *albendazole* (IDA) for clearing microfilaremia (Mf) in individuals with lymphatic filariasis (LF). Although these initial pharmacokinetic and efficacy studies were necessary first steps in the clinical development of IDA, they were not sufficient to justify policy changes necessary for widespread use of this new regimen by national filariasis elimination programs. Processes and procedures that led to the World Health Organization’s (WHO) endorsement of IDA as a mass drug administration (MDA) regimen for LF are reviewed elsewhere in this Supplement. However, the “guideline review process” depended heavily on preliminary results from multicenter studies that were performed to compare the safety, tolerability, and acceptability of IDA versus DA (the two-drug regimen of DEC plus albendazole that was recommended for use for filariasis elimination in countries without co-endemic onchocerciasis or loiasis). Efficacy and tolerability results from those studies have been recently published. Therefore, this paper will focus on practical aspects of the *planning* and *conduct* of the large-scale studies that were so critically important for policy change.

## BACKGROUND

The *Death to Onchocerciasis and Lymphatic Filariasis* (DOLF) Project was established in 2010 as a multi-center consortium for research on selected neglected tropical diseases. ^1^ It was funded by the Bill & Melinda Gates Foundation (BMGF) to conduct clinical trials and community treatment studies to optimize use of approved medicines to accelerate global elimination programs for lymphatic filariasis (LF) and onchocerciasis. Death to Onchocerciasis and Lymphatic Filariasis provides planning and technical support (remote and on-site) to consortium partners in disease-endemic countries.

### Initial clinical trials of IDA.

Death to Onchocerciasis and Lymphatic Filariasis’s first pilot clinical trial of triple drug combination treatment with ivermectin plus diethylcarbamazine and albendazole (IDA) for LF was initiated in 2013 in Papua New Guinea (PNG). ^2^ This trial compared the tolerability and efficacy of IDA (a single oral dose of ivermectin [200 μg/kg] plus DEC [6 mg/kg] plus albendazole [400 mg]) to the two-drug MDA regimen, DA (DEC plus albendazole), then recommended by WHO for PNG and similar countries with LF but without co-endemic onchocerciasis or loiasis. This study had a very dramatic outcome, because it found that that:

a. albendazole had superior efficacy relative to DA for completely clearing *Wuchereria bancrofti* Mf at 12 (and later 24) months after a single treatment dose;
b. the addition of ivermectin to the standard DA regimen did not significantly alter the pharmacokinetic parameters for either DEC or albendazole; and
c. mild to moderate adverse events (AEs) were more common after IDA than after DA, but the treatment was clinically well tolerated. ^2^

A second, larger clinical study of IDA versus DA was initiated in 2014 (also in PNG) that later confirmed that a single dose of IDA was both well tolerated and markedly superior in efficacy to DA (with almost complete clearance of Mf at 12 and 24 months). The DOLF team recognized the potential importance of IDA for the global effort to eliminate LF before the final results from that study or a subsequent study performed in Côte d’Ivoire were available. ^3^^,^ ^4^ They suggested to technical advisors and stakeholders that further clinical development of this extraordinary advance should be accelerated beyond the “normal” pace of research. Key stakeholders included (among others) funding agencies such as the Gates Foundation and the United States Agency for International Development, other research groups, drug donors, and the World Health Organization (WHO). The framework for this accelerated research, outlined in more detail elsewhere in this Supplement, ^5^ focused on identifying and engaging the key decision-makers in this development pathway, defining the specific targets that needed to be reached, assuring availability of resources and providing high-quality technical competence for all aspects of the research. The framework also emphasized that different aspects of the work proceed in parallel when this was feasible rather than in the usual sequential manner.

### Requirements for changing guidelines for LF elimination programs.

World Health Organization’s input was essential for planning the development pathway, because the goal was to identify a treatment regimen that could improve upon WHO’s then-current global health guidelines for eliminating LF. BMGF convened a high-level strategy meeting in Seattle in the summer of 2015 (after the results of the pilot study were known but before the second study was completed). Key decision-makers and stakeholders agreed that the development of IDA was potentially transformational for the LF elimination program. They also agreed that further clinical development should be fast-tracked and focused on meeting requirements for creating new WHO guidelines. This would require assessments of safety, population acceptability, and operational feasibility. Also essential at this time was the funding agency’s agreement to fast-track the necessary research by supporting overlapping (parallel) studies, providing additional critical technical expertise and oversight where needed, and agreeing that available funds could be repurposed toward targets that were of immediate importance to IDA development. Following the Seattle meeting and subsequent planning meetings, DOLF was charged with the task of organizing and implementing the many facets of the research studies necessary to support IDA’s fast-track development.

### Preparation for the studies: Identification of study sites and international partners.

1. **The first challenge was to determine where to conduct the studies.** Considerations included:

a. *Size and scope*. World Health Organization recommends cohort event monitoring with sample sizes sufficient to show that the frequency of serious adverse event (SAEs) (upper 95% confidence interval) after new treatments is less than 0.1%. ^6^ This target required a sample size of 10,000 for IDA and the same number for the DA comparator treatment. The comparator treatment was necessary, because we needed to compare the tolerability of IDA with DA, which rarely causes SAEs after LF treatment. Mirrored studies with DA were also necessary for comparisons of acceptability and efficacy of the two regimens provided as MDA in community settings.
b. *Geographical representation, epidemiological diversity, and disease burden*. Studies were performed in three different WHO regions with varied LF epidemiology (climate, vectors, and prior MDA). Two study sites were treatment-naïve; others had failed to reach WHO LF elimination targets despite many prior rounds of MDA using the recommended DA treatment regimen. One study site had brugian filariasis, which accounts for 5–10% of the global LF burden. This was to be the first use of IDA in a *Brugia*-endemic area.
c. *Areas with significant LF prevalence/persistence*. The DOLF team met with endemic country researchers during site visits or in-person meetings in Europe or the United States. The project commissioned small pilot surveys in several countries to verify LF endemicity at candidate study sites. This verification was important to avoid conducting community studies in areas without significant endemicity.
d. *Partners with proven research capacity*. The DOLF team in St. Louis (DOLF Central) is small and needed to have reliable partners with credibility in their home countries to successfully conduct these complex studies. Project-specific training would be required at all sites, but partners were needed who had prior LF research experience and proven track records. Also necessary was a research infrastructure that could support contracts, fiscal accounting, and compliance with regulatory requirements.

*Five study sites were selected*. Sites in PNG and in a *Brugia*-endemic area in Indonesia were in treatment-naïve areas in countries with high LF burdens and strong prior histories of working on DOLF research projects. Three study sites were in areas with persistent LF despite repeated rounds of MDA. One of these was in India, a high-burden country with a well-recognized LF research center. The second site was in Haiti, a country with the highest LF burden in the Americas that had a research team with significant LF experience. The third site was in Fiji, which was chosen to represent areas with LF transmitted by efficient *Aedes* mosquito vectors.

2. **Robust communication and data management** were essential for this project and depended heavily on the electronic capture of study data (EDC) on portable devices with transfer of encrypted files to a cloud server. System selection was based on results of a landscape analysis performed by an experienced clinical trial consultant. Her analysis considered requirements for rapid reporting of SAEs, features to reduce data entry errors, and manageable costs. Death to Onchocerciasis and Lymphatic Filariasis Central team members had frequent online meetings and e-mail communications with clinical trial consultants, endemic country partners, and a senior program officer at the BMGF. An experienced data manager at DOLF Central worked with the vendor and consultants to tailor data entry, data transfer, and reporting systems to meet requirements for the studies. This included the development of training materials and visits to each study site to train local personnel.

Each study site provided a local designated data manager and data/communication team for data entry, information transfer and synchronization, query resolution, and equipment upkeep. Communication required Internet access via enterprise-quality land connections (India and Haiti) or satellite connections (remote sites in PNG, Fiji, and Indonesia). All sites used barcodes to track participant records and specimens. Each site maintained a separate, confidential “participant key” database to link participant numbers to identifying information such as name and address. Barcode stickers were affixed to clinical samples and paper records such as SAE reports and Mf test and serology results.

3. **Regulatory compliance**. Some common elements applied to all study sites, but there were also specific requirements determined by local conditions and regulations, as indicated in Table 1.
a. *Protocols*. Death to Onchocerciasis and Lymphatic Filariasis Central developed a core research protocol with input from partners and consultants. International partners modified this template according to site-specific requirements. Protocols were reviewed by institutional review boards (IRBs) at Washington University and at research partner institutions. Import permits were required for ivermectin.
b. *Data safety monitoring board (DSMB) and site monitors*. Death to Onchocerciasis and Lymphatic Filariasis Central convened a DSMB to monitor safety for all the participating sites, and some sites also arranged for local DSMBs. Each site had independent site monitoring for Good Clinical Practice (GCP) compliance provided by contract research organizations and a medical monitor for independent assessment of SAEs.
a. *Other documentation and harmonization*. Death to Onchocerciasis and Lymphatic Filariasis Central and clinical trial consultants prepared standard operating procedures (SOPs) and plans for virtually all aspects of the study. Standard operating procedures helped to harmonize activities so that data would be comparable across different study sites. Formal written plans were required to standardize the research activities and to avoid later misunderstandings regarding key aspects of the project such as data management, statistical analysis, study medication storage and accounting, data sharing, and publication policy.
4. **Site preparation by research partners in study countries.** Local research teams were responsible for communication with their national and sub-national health ministries and regulatory authorities. They also hired personnel and made local arrangements for housing and transportation of project staff. Most importantly, local teams were responsible for social mobilization to enhance community participation in their study. This activity varied from site to site, and it generally started long before the first participants were enrolled.
5. **Onsite training for study initiation.** Death to Onchocerciasis and Lymphatic Filariasis Central personnel traveled to each study site and worked with consultants/contractors to provide training on technical aspects of the study to field teams. Training included laboratory procedures, GCP, and use of the electronic data capture and data transfer system. Most sites completed training in 5–7 days. Training also included mock participant interviews for enrollment and for assessment of AEs. The next step of the process was slow-enrollment field visits that provided clinical teams with opportunities to carefully perform all aspects of enrollment and treatment in a small number of houses with supervision by senior observers. This was important, because it helped to iron out wrinkles and identify unanticipated problems or ambiguities. Local political leaders and primary health center personnel sometimes accompanied research teams during slow enrollment. This helped to introduce the study teams to communities and enhance cooperation.
6. **Flexibility.** The original plan was to conduct this study in four sites. The first study was initiated in India in early October 2016, and the studies in Indonesia, Haiti, and PNG followed soon thereafter. A fifth study (in Fiji) was added later to ensure that overall enrollment targets would be reached and to take advantage of a special opportunity to study the effects of MDA with IDA on scabies as well as LF. Each study site experienced growing pains early in the study. There was a learning curve for study procedures, and it took time for disparate groups involved in the work to function smoothly as one team. Other common challenges included poor Internet bandwidth and minor bugs in the EDC system that were promptly resolved by the vendor. Enrollment was more difficult in areas that had already received many rounds of MDA despite special social mobilization efforts. Research teams thought that additional training time would not have corrected the problem. Teams needed flexibility and resourcefulness to overcome challenges.

**Table 1:** DOLF IDA safety and efficacy study sites and regulatory requirements

Features of the study sites and regulatory requirements	Haiti	India	Indonesia	PNG	Fiji
Number of participants enrolled	5,998	8,918	3,926	4,563	3,431
Field research led by 1) MOH; 2) Govt research org; 3) Academic institution(s)	1	2	3	1, 2	1, 3
Number of levels of ethical/regulatory review in country	1	5	1	2	2
Special permit required for importation of study drugs	No	Yes	Yes	Yes	No
Specific, active support by a national minister or higher official	Yes	Yes	No	No	Yes
Financial contribution by country MOH	No	No	No	No	No
Public advocacy by MOH (National, State, Province, District)	P	N, S, D	D	D	P, D
Active support from WHO (Regional and/or Country office)	No	R	No	R, C	R, C
On-site international partner technical support	Yes	Yes	Yes	Yes	Yes
On-site CRO or other contractor(s)	Yes	Yes	No	No	No
Generator required for field studies	Yes	No	Yes	Yes	Yes
Satellite internet required	No	No	Yes	Yes	Yes
Research team prior experience with large-scale clinical trials	Limited	Yes	Yes	Limited	Limited
National research team required to travel by air and/or sea to study sites	No	Air	Air	Air	Air and Sea

DOLF = Death to Onchocerciasis and Lymphatic Filariasis; IDA = ivermectin plus DEC and albendazole.

### Studies performed to assess the acceptability of IDA: rationale and benefits.

The initial sense of urgency for the IDA safety and efficacy studies reflected broad agreement that IDA could be a game changer for LF elimination in eligible endemic countries. However, the new treatment would have little value if it was not acceptable to target populations. Would the additional pills or fears of AEs pose an added barrier to compliance? ^7^ Would communities appreciate the ancillary benefits that ivermectin provides? Such issues could affect compliance with IDA even if the community studies found that it was safe and effective. Would the global research community want to pause to investigate IDA acceptability *after* completion of community safety studies, or would programs rush to use the new regimen?

After much discussion, the DOLF team and the BMGF decided to include acceptability studies as part of the community safety and efficacy trials in all five countries. The acceptability studies would follow the IDA/DA drug administration by several months to allow some separation between the clinical trial and the acceptability research. ^8^ Participants were randomly selected from the same participant list used by the safety trial. The concept of MDA acceptability had not been formally addressed in prior NTD research studies. Acceptability has been assumed to be present when people ingest MDA drugs. However, as 100% of the people enrolled in the initial, closely monitored clinical trials had swallowed either DA or IDA tablets, it was impossible to use tablet ingestion as an indicator of acceptability. The acceptability research team turned to the field of educational and behavioral interventions to develop a new measure of MDA acceptability. ^9^ This measure was derived from the Intervention Rating profile tool together with validated questions from other surveys to develop a framework for the acceptability studies. ^10^^–^ ^12^ Use of a uniform tool across five different contexts and multiple languages required precise translation of the instruments. Any variation would reduce comparability and prohibit the team from reliably measuring inter-country differences in acceptability of IDA versus DA. Also, the timing of the acceptability studies had to be coordinated with the tolerability and efficacy trial schedules; delays in the safety studies affected the acceptability study timelines. Regular communication between various members of the research team was essential for planning and adaptation.

Locally based research partners were identified to lead acceptability studies in each study site. These teams highlighted cultural, linguistic, logistic, and contextual issues that could affect both the study and IDA acceptability in their countries. Results from the global acceptability study showed that “country” was the most significant driver for differences in acceptability at the different sites. These differences may have been related to prior experiences with MDA and other healthcare services.

Linking acceptability studies to the safety and efficacy trial was advantageous on many levels. First, the finding that study communities considered DA and IDA to be equally acceptable was consistent with MDA safety data from the same localities. Second, it generated evidence from five sites demonstrating that the increased number of tablets was not a barrier to IDA uptake. Third, results of the study led to key recommendations for the global LF program on how to improve MDA compliance with *either* DA or IDA. The study also showed that linking acceptability studies to clinical research created efficiencies (in both time and cost) for assessments of new treatments. Subsequent large-scale rollouts of IDA in early adopter countries applied learnings from these acceptability studies as they planned to introduce the new MDA regimen. Finally, unbeknownst to the DOLF research team at the start of their safety and efficacy studies, evidence of acceptability turned out to be a key component required for the WHO policy change process. ^13^

## RESULTS AND DISCUSSION

### Conduct of the studies and communication of results to WHO.

Important, site-specific information is summarized in Table 1. All of the studies proceeded rapidly once they started. Although this speed was due in part to extensive planning and preparation, the talented and dedicated field research teams deserve kudos for their hard work under difficult conditions. Country-specific summaries are presented in a companion article in this Supplement.

To accommodate the fast-tracked timelines, data managers and statisticians had to work rapidly under time pressure to clean, organize, and analyze the data. Complex acceptability studies based on mixed methods approaches were completed shortly after MDA so that findings could be shared with WHO along with the tolerability results.

Death to Onchocerciasis and Lymphatic Filariasis was able to share preliminary results from these studies with WHO in Q1 2017, when treatment and AE monitoring data were available for more than 20,000 participants in four countries. It is unusual for researchers to share raw data from clinical trials for independent analysis before enrollment is completed. This happened less than 18 months after DOLF had received a “Go” signal from the BMGF to conduct and coordinate these studies. The WHO LF Guidelines Development Group (GDG) first reviewed tolerability data from the study in May of 2017, and preliminary, favorable results from *acceptability* studies conducted in three countries were also presented at that time. Consideration of both types of data made sense, because acceptability assessments are essential components for policy changes at WHO. Thus, the accelerated development process was possible not only because of the high degree of coordination and communication between DOLF, the BMGF, and WHO, but also because of WHO’s extraordinary efforts to coordinate and accelerate the review process through its offices in Geneva, regional headquarters, and countries (as described elsewhere in this Supplement). World Health Organization published a guideline on alternative MDA regimens for LF elimination in November 2017 that summarized the review process and recommended use of IDA in certain settings. ^14^

### Remaining challenges on the road to impact.

In 2019, WHO reported that almost 50 million IDA treatments had been distributed in 11 countries, ^15^ and it is likely that more than 100 million treatments were distributed by the end of 2020 (mostly in India) since WHO endorsed IDA use for LF elimination in late 2017. However, IDA use has not yet approached the *yearly* 100 million doses that were enabled by Merck’s commitment for the expanded donation of ivermectin. There are many reasons for this—some technical (e.g., time required for countries to modify drug policy and acquire ivermectin, lack of clear guidance on when to stop MDA after IDA, and COVID-induced delays and shortages), and some more strategic or programatic. ^16^^–^ ^19^ Indeed, some countries believe that they are already on track to eliminate LF in the near future with the older two-drug MDA regimens of IA or DA; momentum and resistance to change certainly play a role in other countries. Programs that had already had difficulty delivering two-drug MDA have also been slow to roll out IDA, and it is clear that IDA alone cannot fix underperforming or underfinanced LF elimination programs. Ongoing implementation research projects in several countries should provide further insight on how IDA introduction can be linked to best practices that improve MDA operations through microplanning, active monitoring, and targeted social mobilization.

### Lessons for other clinical development programs for novel global health interventions.

The IDA clinical development story was unusual. Although the new regimen being tested was a combination of approved drugs with long track records and donation programs, some lessons from this experience should apply to other clinical development programs that aim to change policy.

a. Early stakeholder involvement was essential to promote communication between researchers, donors (medicines and resources), and implementers so that they could work together toward the shared goal of policy change to accelerate LF elimination.
b. Although the project had strong financial support, money alone would not have ensured success. This project benefitted from genuine engagement of experienced leaders who provided advocacy and convening power to cut through red tape in countries and international organizations.
c. The project was built on a foundation of preexisting scientific and financial relationships. It also benefitted from strong central management, aligned protocols, and technologies such as satellite internet and electronic data capture for data management and transfer.
d. The project benefited from coordinated activity by different partners who worked in parallel toward a common goal (*widespread adoption of a new MDA regimen to accelerate LF elimination*). This was a key factor that accelerated the transition from research to policy change.
e. The DOLF team benefitted from early input regarding WHO’s review process and the types and amounts of data that would be required to support policy change. This made it clear to the research team and principal donors that the program’s “pivotal study” had to be large, carefully planned, and conducted with a high professional standard (“go big or go home”). It also had to have proven acceptability in target populations.
